# Refractory Gastric Outlet Obstruction Due to a Duodenal Stricture in the Setting of a Cholecystoduodenal Fistula

**DOI:** 10.7759/cureus.76942

**Published:** 2025-01-05

**Authors:** Natalie Mesa, Deep Vakil, Himani Bhatt, Megan Shumway, Maxine Garcia, Kumar Jayant, Omar Llaguna, Christopher J Gannon

**Affiliations:** 1 Department of Surgery, Florida International University, Herbert Wertheim College of Medicine, Miami, USA; 2 Department of Surgery, Memorial Healthcare System, Hollywood, USA; 3 Department of Surgical Oncology, Memorial Healthcare System, Hollywood, USA

**Keywords:** bilioenteric fistula, cholecystoduodenal fistula, duodenal stricture, refractory gastric outlet obstruction, surgical intervention

## Abstract

Gastric outlet obstruction (GOO) is a clinical condition in which an underlying disease process leads to a lack of gastric emptying. The presentation and management of GOO due to a variety of benign and malignant causes have been researched; however, limited medical literature exists on the presentation and management of refractory GOO caused by a duodenal stricture secondary to a cholecystoduodenal fistula. This case report highlights the diagnostic challenges and the significance of timely intervention.

This case of a 66-year-old female with GOO refractory to multiple endoscopic interventions demonstrates the presentation and management of such a rare cause of GOO and highlights the importance of surgical consultation in persistent GOO.

The etiology of GOO varies widely, requiring a range of treatments from medical management to surgery. This case underscores the importance of identifying the cause to ensure effective treatment. Cholecystoduodenal fistulas are rare but significant, often necessitating surgical intervention when endoscopic procedures fail. Our patient had a duodenal stricture due to a cholecystoduodenal fistula, indicating the necessity for surgical consultation.

## Introduction

Gastric outlet obstruction (GOO) is a clinical condition in which there is mechanical blockage of gastric emptying, generally in the distal stomach, pyloric channel, or duodenum. The differential consists of a range of benign or malignant etiologies due to a motility disorder. Benign causes include gastric or duodenal ulcers, polyps, anastomotic strictures, pancreatitis, Crohn’s disease, and adhesions. Malignant causes include cancer of the stomach, duodenum, pancreas, hepatobiliary system, gastrointestinal stromal tumor, and lymphoma. Historically, peptic ulcer disease was the primary cause of GOO. The widespread use of *Helicobacter pylori* treatment and proton pump inhibitors has greatly reduced the prevalence of peptic ulcer disease, thereby changing the most common causes of GOO. Currently, malignancy is the leading cause of GOO, with pancreatic cancer being the most common in Western countries and gastric cancer in India and other South Asian countries [1].

Bilioenteric fistulas are atypical connections that form spontaneously between the biliary system and the gastrointestinal tract. Biliary-enteric fistulas are an uncommon complication of cholelithiasis, occurring at a rate of 0.5% to 0.9% [2]. These fistulas can result in a range of clinical complications and, in some cases, can pose a life-threatening risk to the patient.

There is literature describing the presentation and management of GOO due to the causes stated above [3,4]. However, to our knowledge, there are no reports on the presentation and management of GOO due to duodenal stricture secondary to cholecystoduodenal fistula. This case report was written in line with the Surgical Case Report (SCARE) Guidelines [5].

## Case presentation

The patient is a 66-year-old female with a two-year history of chronic cholecystitis and two months of nausea, emesis, and nonspecific abdominal pain in the setting of GOO due to duodenal stricture of undetermined etiology. Over a two-month period, she underwent multiple unsuccessful endoscopic interventions including esophagogastroduodenoscopy (EGD) with attempted dilations and failed stent placement. She ultimately required a liquid diet during this time. She later presented to the emergency department with persistent symptoms, new-onset intolerance of liquids, and a 10-pound weight loss. On the physical exam, vital signs were within normal limits and the abdomen was soft, non-distended, and non-tender with no rebound, guarding, or rigidity. Lab results were unremarkable. She was admitted for further evaluation and subsequently underwent EGD with duodenal stricture dilation and endoscopic ultrasound with biopsies.

**Figure 1:**
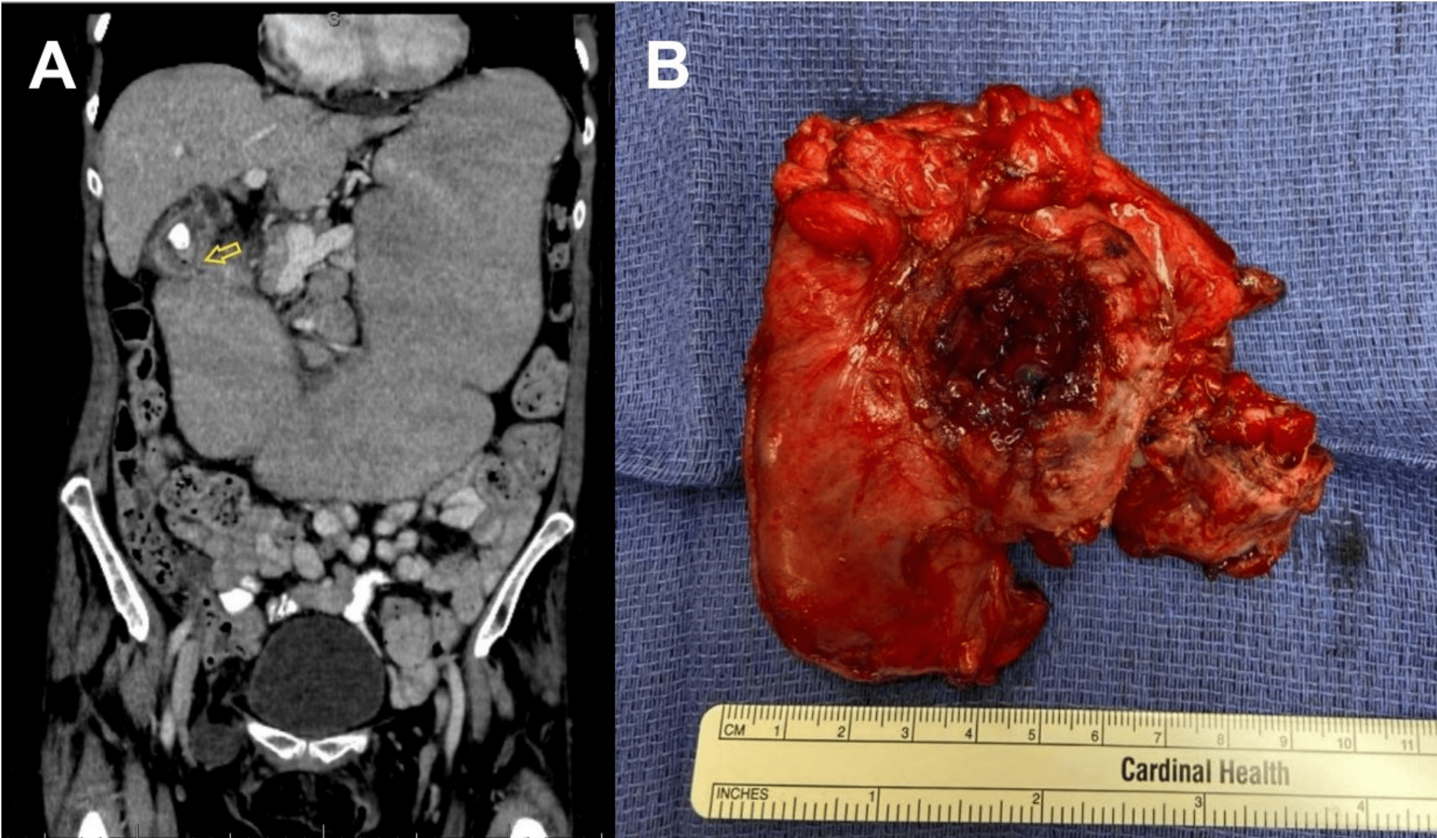
CT of the abdomen and pelvis and intraoperative findings A) Arrow demonstrating retroperitoneal perforation with air and fluid around the duodenum and the cholecystoduodenal fistula on CT of the abdomen and pelvis. B) Intraoperative findings: gallbladder adhered to the duodenum at the site of the stricture and fistula.

The following day, the patient developed severe epigastric abdominal pain with tenderness, voluntary guarding, and a leukocytosis of 16.7. The patient had imaging studies performed prior to her EGD, including a magnetic resonance cholangiopancreatography (MRCP) and a right upper quadrant ultrasound. The MRCP demonstrated wall thickening of the gallbladder and adjacent duodenum, and the ultrasound demonstrated a 3-centimeter gallstone. Given her continued hemodynamic stability, she underwent an urgent computed tomography (CT) of the abdomen and pelvis, which revealed a retroperitoneal perforation with air and fluid around the duodenum and the presence of a cholecystoduodenal fistula (Figure 1A). She was converted to nil per os (NPO), a nasogastric tube was placed, and empiric intravenous antibiotics were initiated. An urgent surgical consult was placed for further management. The patient was taken emergently to the operating room for an exploratory laparotomy where the gallbladder with the gallstone was found to be inseparable from the first and proximal second portion of the duodenum (Figure 1B) at the site of her previously identified GOO. She underwent en bloc distal gastrectomy, proximal duodenectomy, cholecystectomy, antecolic loop gastrojejunostomy, and retroperitoneal abscess drainage.

The patient underwent a normal postoperative course. By postoperative day nine, the patient was tolerating a regular diet and had a return of bowel function. She was subsequently discharged to a skilled nursing facility. Final pathology revealed an inflammatory cholecystoduodenal fistula. The patient was seen at follow-up ten months post-op in the clinic. She had been tolerating a regular diet without any gastrointestinal symptoms.

## Discussion

The etiology of GOO has a broad differential with a range of management options including medical management, endoscopic intervention, and surgery depending on the diagnosis. The current case highlights the importance of determining the etiology of the GOO to provide effective treatment. Bilioenteric fistulas are exceedingly rare and their distributions are as follows: cholecystoduodenal (60%), cholecystocolic (15%), cholecystogastric (5%), and choledochoduodenal (5%) [6]. If present, they more so occur as a complication of a cholecystectomy, which our patient did not undergo, or secondary to peptic ulcers or neoplasia [7]. Given the range of clinical complications, a surgical consultation is warranted to evaluate and determine the optimal intervention for better health outcomes.

The initial hospital management of GOO has many similarities regardless of the etiology. Patients receive a nasogastric tube for gastric decompression, fluids and electrolytes for resuscitation, parenteral proton pump inhibitors, and medications for pain and nausea as needed. The workup to determine the etiology includes plain radiography, CT or magnetic resonance imaging, and upper endoscopy with biopsy. Pneumobilia is a common finding preoperatively that can indicate the presence of an internal biliary fistula, which was not evident in our patient [7]. If there is a concern for malignancy, endoscopic ultrasonography may also be useful. Gastroenterology is consulted to be on the care team.

Once the etiology is determined, it is treated with medical management and endoscopic or surgical intervention. Medical management consists of discontinuing the use of nonsteroidal anti-inflammatory drugs, treating an underlying *H. pylori* infection if present, and appropriate oncological care for malignant causes. In general, for benign causes of GOO, endoscopic dilation is the main intervention. Our patient failed her initial EGD with dilation and the literature suggests that the need for two or more dilations may predict the need for surgery [8]. Patients with a malignant cause may benefit from endoscopic stenting. For patients who fail to improve with medical therapy and endoscopic interventions, surgery can be considered. In this case, the patient failed to improve despite multiple endoscopic procedures because the underlying etiology was a duodenal stricture secondary to a cholecystoduodenal fistula. The fistula was contributing to the narrowing of the duodenum. Additionally, our patient’s recurrent episodes of cholecystitis were causing ongoing inflammation and scarring, worsening the stricture over time. While endoscopic intervention provided temporary relief, the underlying pathophysiology remained resulting in resistance to further endoscopic treatment, thus requiring surgical intervention.

Surgical options include laparoscopic versus open gastrojejunostomy to relieve the obstruction, oncological procedures for malignant causes, or percutaneous gastrostomy for gastric decompression with the placement of a jejunal feeding tube [9]. The latter is for patients who are too high risk for other surgeries and need palliative care. In recent years, advancements in endoscopy have led to endoscopic ultrasound-guided gastroenterostomy (EUS-GE). However, there is no current significant data that suggests EUS-GE is at least as safe and efficacious as surgery [9]. The recommended treatment for cholecystoduodenal fistula is cholecystectomy along with the closure of the fistula communication, and studies suggest that this repair can be performed via laparoscopy or open [7]. A study conducted by Angrisani et al. comparing laparoscopic and open surgery discovered that there were no significant differences in the rates of intraoperative and postoperative complications between the two methods [10]. Our patient underwent an open repair with an uncomplicated postoperative course.

The current case highlights that patients with GOO who have persistent significant symptoms despite multiple endoscopic procedures should be assessed with an expanded working differential and a surgical consultation may be warranted. In patients with chronic cholecystitis and gallstones 3 cm or larger, cholecystoduodenal fistula can be on the differential.

## Conclusions

In conclusion, this rare case illustrates the complexity of managing refractory GOO caused by a duodenal stricture secondary to a cholecystoduodenal fistula. It exemplifies the diagnostic challenges and the importance of timely surgical intervention when endoscopic approaches are insufficient. Identifying the underlying etiology is crucial for guiding appropriate treatment and ensuring successful outcomes.
